# Assessment of Respiratory Function in Infants and Young Children Wearing Face Masks During the COVID-19 Pandemic

**DOI:** 10.1001/jamanetworkopen.2021.0414

**Published:** 2021-03-02

**Authors:** Riccardo Lubrano, Silvia Bloise, Alessia Testa, Alessia Marcellino, Anna Dilillo, Saverio Mallardo, Sara Isoldi, Vanessa Martucci, Maria Sanseviero, Emanuela Del Giudice, Concetta Malvaso, Donatella Iorfida, Flavia Ventriglia

**Affiliations:** 1 Pediatric and Neonatology Unit, Maternal and Child Department, Sapienza University of Rome, Polo Pontino, Rome Italy

## Abstract

**Importance:**

Face masks have been associated with effective prevention of diffusion of viruses via droplets. However, the use of face masks among children, especially those aged younger than 3 years, is debated, and the US Centers for Disease Control and American Academy of Physicians recommend the use of face mask only among individuals aged 3 years or older.

**Objective:**

To examine whether the use of surgical facial masks among children is associated with episodes of oxygen desaturation or respiratory distress.

**Design, Setting, and Participants:**

This cohort study was conducted from May through June 2020 in a secondary-level hospital pediatric unit in Italy. Included participants were 47 healthy children divided by age (ie, group A, aged ≤24 months, and group B, aged >24 months to ≤144 months). Data were analyzed from May through June 2020.

**Interventions:**

All participants were monitored every 15 minutes for changes in respiratory parameters for the first 30 minutes while not wearing a surgical face mask and for the next 30 minutes while wearing a face mask. Children aged 24 months and older then participated in a walking test for 12 minutes.

**Main Outcomes and Measures:**

Changes in respiratory parameters during the use of surgical masks were evaluated.

**Results:**

Among 47 children, 22 children (46.8%) were aged 24 months or younger (ie, group A), with 11 boys (50.0%) and median (interquartile range [IQR]) age 12.5 (10.0-17.5) months, and 25 children (53.2%) were aged older than 24 months to 144 months or younger, with 13 boys (52.0%) and median (IQR) age 100.0 (72.0-120.0) months. During the first 60 minutes of evaluation in the 2 groups, there was no significant change in group A in median (IQR) partial pressure of end-tidal carbon dioxide (Petco_2_; 33.0 [32.0-34.0] mm Hg; *P* for Kruskal Wallis = .59), oxygen saturation (Sao_2_; 98.0% [97.0%-99.0%]; *P* for Kruskal Wallis = .61), pulse rate (PR; 130.0 [115.0-140.0] pulsations/min; *P* for Kruskal Wallis = .99), or respiratory rate (RR; 30.0 [28.0-33.0] breaths/min; *P* for Kruskal Wallis = .69) or for group B in median (IQR) Petco_2_ (36.0 [34.0-38.0] mm Hg; *P* for Kruskal Wallis = .97), Sao_2_ (98.0% [97.0%-98.0%]; *P* for Kruskal Wallis = .52), PR (96.0 [84.0-104.5] pulsations/min; *P* for Kruskal Wallis test = .48), or RR (22.0 [20.0-25.0] breaths/min; *P* for Kruskal Wallis = .55). After the group B walking test, compared with before the walking test, there was a significant increase in median (IQR) PR (96.0 [84.0-104.5] pulsations/min vs 105.0 [100.0-115.0] pulsations/min; *P* < .02) and RR (22.0 [20.0-25.0] breaths/min vs 26.0 [24.0-29.0] breaths/min; *P* < .05).

**Conclusions and Relevance:**

This cohort study among infants and young children in Italy found that the use of facial masks was not associated with significant changes in Sao_2_ or Petco_2_, including among children aged 24 months and younger.

## Key Points

**Question:**

Are surgical masks associated with episodes of oxygen desaturation or respiratory distress among children?

**Findings:**

In this cohort study of 47 infants and young children in Italy, wearing surgical face masks for 30 minutes was not associated with changes in respiratory parameters or clinical signs of respiratory distress.

**Meaning:**

These findings suggest that the use of surgical masks among children may be promoted during the coronavirus disease 2019 pandemic, especially in view of the reopening of schools.

## Teaser

This cohort study examines whether the use of surgical face masks among children was associated with changes in respiratory function or signs of respiratory distress.

## Introduction

During the coronavirus disease 2019 (COVID-19 virus) pandemic, a large amount of useful data has provided a better understanding of virus behavior. Confronting the experiences of individual countries, we can evaluate the efficacy associated with preventive measures enforced during lockdowns. Based on these experiences, governments and public health agencies are now developing recommendations aimed at containing a possible peak of infection, especially in view of the reopening of schools.

Currently, there is a consensus that the main route of transmission of COVID-19 virus is via droplets, although there is not complete agreement on details (eg, span, size, and virus concentration).^1,2,3,4,5,6^ In fact, discrepancies have been observed in the general public and community settings in attitudes about containment measures, with different national guidelines proposed to slow the spread of COVID-19.^7^

Another issue that frequently arises in the debate is the role of individuals who have the disease but no symptoms in spreading the infection; 2 studies^8,9^ found a high rate of positive pharyngeal molecular test results among individuals without symptoms, and that may become an important source of contagion in the pandemic.^10,11^ Children have also been found to commonly carry the disease without symptoms.^12,13^ This is more difficult to identify among children, because they commonly exhibit milder symptoms of COVID-19 compared with adults.^14^

However, the role of children in the spread of severe acute respiratory syndrome coronavirus 2 (SARS CoV-2) infection is still debated. Five studies^15,16,17,18,19^ have found significantly decreased transmission of SARS-CoV-2 transmission associated with children compared with adults, with a susceptibility to infection among children that is about half of that of adults and a decreased likelihood of developing symptoms. However, 5 other studies^14,20,21,22,23^ found that children can carry high levels of virus in their upper airways, particularly early in an acute SARS-CoV-2 infection, even if it is a mild infection or asymptomatic infection. Because of these findings on asymptomatic carrying, the main measures proposed for preventing the spread of SARS-CoV-2 are social distancing, handwashing, and mask-wearing,^24^ not only for health care workers, but also for the general population. The association of surgical face masks with effective prevention of transmission was already found during the 2003 SARS virus epidemic.^25^ Studies^26,27,28^ published in 2020 found that the use of face masks is also associated with preventing transmission from individuals without symptoms. Furthermore, a mathematical model^29^ has further suggested the protective role of masks, finding a linear association between the use of masks and a decreased rate of spread of COVID-19 infection.

Adverse outcomes associated with wearing face masks have been described in adult patients.^30,31^ Among children, the US Centers for Disease Control and Prevention (CDC)^32^ and the American Academy of Pediatrics (AAP)^33^ do not recommend the use of face masks among individuals aged younger than 3 years. These organizations also recommend special precautions for children with severe cognitive or respiratory impairments. Children in these groups also experience problems with the use of face masks because poor motor skills and coordination may make it difficult to remove the mask. In addition to poor compliance with mask wearing expected in a pediatric population, these indications for the use of face masks among children aged younger than 3 years or children with disabilities are often misinterpreted and are associated with widespread public distrust of face mask use among children.

The evidence for adverse outcomes associated with face mask use among children is poor, and the benefits of preventing individuals without symptoms or with mild symptoms from spreading the infection is obvious. We therefore decided to examine the potential associations of the use of surgical face masks with changes in respiratory function among children, including those aged younger than 2 years.

## Methods

This cohort study was approved by the Pediatric Unit of Santa Maria Goretti Hospital, Latina—Sapienza University of Rome department institutional review board. For each child included in the study, informed written consent was obtained from available parents or caregivers. The study protocol conformed to the ethical guidelines of the 1975 Declaration of Helsinki as revised in 2000.^34^ This report followed the Strengthening the Reporting of Observational Studies in Epidemiology (STROBE) reporting guideline for cohort studies.

The study’s primary aim was to determine if the use of surgical mask among younger children was associated with changes in respiratory function, as measured by partial pressure of end-tidal carbon dioxide (Petco_2_), oxygen saturation (Sao_2_), pulse rate (PR), and respiratory rate (RR), or with manifestation of clinical signs of respiratory distress. We monitored Petco_2_ using a microstream system. The secondary aim was to determine if the use of masks in this population was associated with a decrease in the perfusion index (PI).

From May 2020 to June 2020, we enrolled 47 healthy children (aged 4 to 144 months). Exclusion criteria were lung or cardiac disease, neuromuscular disorders, use of medications that could be associated with changes in the parameters examined, and age older than 144 months. Participants were divided into 2 groups: children aged older than 4 months to 24 months or younger (ie, group A) and children aged older than 24 months to 144 months or younger (ie, group B).

For every child admitted to the study, we implemented the following steps: Two days before the study started, a pediatrician (A.T.) examined the child to verify the individual’s state of wellness and to adjust the surgical face mask to fit comfortably; in particular, we tucked the edges into the inner surface and sewed them. We used single-use surgical masks (BYD Precision Manufacture) with 3 layers (ie, an outer layer of polypropylene spunbonded nonwoven fabric, a middle layer of polypropylene melt-blown nonwoven fabric, and an inner layer of polypropylene spunbonded nonwoven fabric), ear loops consisting of a polyester and nylon spandex blend, and a nasal clip consisting of metal wire with plastic covering. Then, doctors educated the parents about the correct way for the children to wear and take off the mask. After that, to achieve the best compliance from the child, parents encouraged the children to wear the mask at home as a game for short periods. Parents also showed children a promotional video sponsored by the Italian Society of Pediatric Emergency and Urgent Medicine and produced by the Italian Emergency Health Society to help children to get accustomed to surgical face masks.^35^ On the evaluation day, parents and doctors also wore surgical masks to help obtain maximal compliance from the children.

The test consisted of two 30-minutes sessions, the first without a mask and the second with the surgical face mask in place. In both sessions, the children were encouraged to engage in their usual play activity. For group B, a third session was included, which consisted of walking for 12 minutes back and forth along a 40-m corridor while still wearing the mask. The instructions given for this last session were similar to those for the 12-minute walking test.^36^ We used walking as a strain test because it better reflects a normal physical activity compared with other game activities. The children were encouraged by the supervising doctor to walk fast, and the distance traveled was recorded.

During the study, every child was connected to a Masimo patient-monitoring system (Rad-97 with NomoLine Capnography) to record Petco_2_ (measured in millimeters of mercury), Sao_2_ (measured as percentage), PR (measured as pulsations/min), and PI (measured as percentage). The RR (measured as breaths/min) was detected manually by an observing doctor (A.T.). All parameters were recorded every 15 minutes: at 15 minutes from the start, 30 minutes, 45 minutes, and 60 minutes. For group B, we did an additional evaluation during the 12-minute walking test, at 72 minutes from the start of the evaluation. The supervising physician (A.T.) was in charge of detecting any signs of respiratory distress, such as retractions, abnormal skin color, and use of accessory muscles.

### Statistical Analysis

For all parameters considered in the study, the approximation to normal of the distribution of the population was tested by Kolmogorov-Smirnov 1-sample test and statistics for kurtosis and symmetry. As results were asymmetrically distributed, nonparametric tests were used. Data are expressed as median and interquartile range (IQR). We used the Kruskal-Wallis nonparametric 1-way analysis of variance to examine the changes of each parameter within the subgroups; the null hypothesis was that the groups for the same parameter all came from the same distribution. Then, to exclude the eventuality of a type 1 error between groups for the same parameter, we determined the *P* value associated with each of the comparisons using the Steel-Dwass method as a nonparametric test for multiple comparisons.

Data were analyzed from May through June 2020 using JMP statistical software version 14.3.0 (SAS Institute). *P* values were 2-sided, and *P* < .05 was considered statistically significant.

## Results

A total of 47 children were included in the analysis. Group A consisted of 22 children (46.8%), with 11 (50.0%) boys and median (IQR) age 12.5 (10.0-17.5) months; 2 children (1 boy, aged 8 months, and 1 girl, aged 11 months) dropped out because of intolerance of the mask. Group B consisted of 25 children (53.2%), with 13 boys (52.0%) and median (IQR) age 100.0 (72.0-120.0) months (Table 1).

**Table 1.** Demographic Characteristics of Participants

Characteristic	Participants by group, median (IQR)	
	Group A (n = 22)	Group B (n = 25)
Boys, No. (%)	11 (50.0)	13 (52.0)
Age, mo	12.5 (10.0-17.5)	100.0 (72.0-120.0)
Weight, kg	10.2 (9.0-11.7)	38.0 (25.6-42.8)
Height, cm	74.0 (70.6-81.8)	133.5 (123.3-142.4)
BMI	17.4 (16.4-18.9)	20.3 (15.9-22.4)

Abbreviations: BMI, body mass index (calculated as weight in kilograms divided by height in meters squared); IQR, interquartile range.

The analysis of the readings of Sao_2_, Petco_2_, PR, RR, and PI at set recording times of the study in both groups are provided in Table 2. There was no statistically significant change during the study period in median (IQR) Sao_2_ in group A (at 15 min: 98.0% [97.3%-98.0%]; at 30 min: 98.0% [98.0%-99.0%]; at 45 min: 98.0% [97.0%-98.8%]; at 60 min: 98.0% [97.5%-98.0%]; *P* for Kruskal Wallis = .61) or group B [at 15 min: 98.0% [98.0%-98.0%]; at 30 min: 98.0% [97.0%-98.0%]; at 45 min: 98.0% [97.5%-98.0%]; at 60 min: 98.0% [97.0%-98.0%]; after walking test: 98.0% [97.0%-98.0%]; *P* for Kruskal Wallis = .52); median (IQR) Petco_2_ in group A (at 15 min: 33.0 [32.3-35.0] mm Hg; at 30 min: 33.5 [32.3-34.8] mm Hg; at 45 min: 33.0 [32.0-34.0] mm HG; at 60 min: 32.5 [32.0-34.0] mm Hg; *P* for Kruskal Wallis = .59) or group B (at 15 min: 37.0 [34.0-39.0] mm Hg; at 30 min: 36.0 [34.0-38.0] mm Hg; at 45 min: 36.0 [35.0-37.5] mm Hg; at 60 min: 36.0 [34.0-38.0] mm Hg; after walking test: 36.0 [35.0-37.5] mm Hg; *P* for Kruskal Wallis = .97); or median (IQR) PI in group A (at 15 min: 3.5% [2.6%-4.5%)]; at 30 min: 2.9% [2.6%-4.3%]; at 45 min: 3.8% [2.6%-4.8%]; at 60 min: 3.6% [2.6%-4.5%]; *P* for Kruskal Wallis = .89) or group B (at 15 min: 4.6% [3.0%-5.8%]; at 30 min: 4.3% [2.9%-6.5%]; at 45 min: 4.1% [2.6%-6.2%]; at 60 min: 4.3% [2.8%-5.9%]; after walking test: 3.5% [2.7%-5.0%]; *P* for Kruskal Wallis = .77) (Table 2).

**Table 2.** Respiratory Parameter Measures

Parameter	Participants by group, median (IQR)									
	Group A					Group B				
	At 15 min	At 30 min	At 45 min	At 60 min	At 15 min		At 30 min	At 45 min	At 60 min	After walking test
Sao_2_, %	98.0 (97.3-98.0)	98.0 (98.0-99.0)	98.0 (97.0-98.8)	98.0 (97.5-98.0)	98.0 (98.0-98.0)		98.0 (97.0-98.0)	98.0 (97.5-98.0-)	98.0 (97.0-98.0)	98.0 (97.0-98.0-)
Petco_2_, mm Hg	33.0 (32.5-35.0)	33.5 (32.3-34.8)	33.0 (32.0-34.0)	32.5 (32.0-34.0)	37.0 (34.0-39.0)		36.0 (34.0-38.0)	36.0 (35.0-37.5)	36.0 (34.0-38.0)	36.0 (35.0-37.5)
PR, pulsations/min	128.5 (113.5-140.0)	128.5 (110.5-140.0)	130.0 (118.5-140.0)	130.0 (116.3-140.0)	90.0 (84.0-103.5)		91.0 (80.0-97.0)	90.0 (85.0-98.5)	99.0 (83.0-102.0)	105.0 (100.0-115.0)
RR, breaths/min	30.0 (28.0-31.5)	31.0 (28.0-33.0)	30.0 (26.5-33.8)	31.0 (26.5-32.0)	20.0 (17.5-24.0)		21.0 (19.0-24.5)	22.0 (20.0-25.0)	24.0 (19.0-26.0)	26.0 (24.0-29.0)
PI, %	3.5 (2.6-4.5)	2.9 (2.4-4.3)	3.8 (2.6-4.8)	3.6 (2.6-4.5)	4.6 (2.9-5.8)		4.3 (2.9-6.5)	4.1 (2.6-6.2)	4.3 (2.8-5.9)	3.5 (2.7-5.0)

Abbreviations: IQR, interquartile range; Petco_2_, partial pressure of end-tidal carbon dioxide; PI, perfusion index***;*** PR, pulse rate; RR, respiratory rate; Sao_2_, oxygen saturation.

In addition, there was no significant change over the study period for group A in median (IQR) PR (at 15 min: 128.5 [113.5-140.0] pulsations/min; at 30 min: 128.5 [110.5-140.0] pulsations/min; at 45 min: 130.0 [118.5-140.0] pulsations/min; at 60 min: 130.0 [84.0-103.5] pulsations/min; *P* for Kruskal Wallis = .99) or RR (at 15 min: 30.0 28.0-[31.5] breaths/min; at 30 min: 31.0 [28.0-33.0] breaths/min; at 45 min: 30.0 [26.5-33.8] breaths/min; at 60 min: 31.0 [26.5-32.0] breaths/min; *P* for Kruskal Wallis = .69).

After the walking test in group B, there was a significant increase in median (IQR) PR (at 15 min: 90.0 [84.0-103.5] pulsations/min; at 30 min: 91.0 [85.0-98.5] pulsations/min; at 45 min: 90.0 [85.0-98.5] pulsations/min; at 60 min: 99.0 [83.0-102.0] pulsations/min; after walking test: 105.0 [100.0-115.0] pulsations/min; *P* for Kruskal Wallis = .002) and median (IQR) RR (at 15 min: 20.0 [17.5-24.0] breaths/min; at 30 min: 21.0 [19.0-24.5] breaths/min; at 45 min: 22.0 [20.0-25.0] breaths/min; at 60 min: 24.0 [19.0-26.0] breaths/min; after walking test: 26.0 [24.0-29.0] breaths/min; *P* for Kruskal Wallis = .002). In the Steel-Dwass test, there was a significant increase after the walking test compared with all previous time points for PR (at 15 min: *P* < .04; at 30 min: *P* < .01; at 45 min: *P* < .01; at 60 min: *P* < .02) and RR (at 15 min: *P* < .001; at 30 min: *P* < .002; at 45 min: *P* < .01; at 60 min: *P* < .05).

In group A, median (IQR) value for all parameter readings from 15 minutes after study start to 60 minutes after study start was 33.0 (32.0-34.0) mm Hg for Petco_2_, 98.0% (97.3%-99.0%) for Sao_2_, 130.0 (115.0-140.0) pulsations/min for PR, 30.0 (28.0-33.0) breaths/min for RR, and 3.5% (2.5%-4.5%) for PI. In group B, the median (IQR) value for all readings was 36.0 (34.0-38.0) mm Hg for Petco_2_, 98.0% (97.0%-98.0%) for Sao_2_, 96.0 (84.0-104.5) pulsations/min for PR, 22.0 (20.0-25.0) breaths/min for RR, and 4.3% (2.8%-5.8%) for PI.

The mean (IQR) distance traveled by children during the walk test was 808.0 (920.0-557.8) m. Throughout the duration of the study, no child showed clinical signs of respiratory distress.

## Discussion

This cohort study found that use of surgical masks among children was not associated with episodes of oxygen desaturation or the development of clinical signs of respiratory distress during a walking test. We monitored Petco_2_ using a microstream system because this method is associated with greater accuracy. Because there is a temporal gap between the reduction of arterial oxygen pressure (ie, Pao_2_) and its detection in the arterial blood,^37^ this technical improvement may allow clinicians to more promptly detect a decrease in alveolar ventilation, a signal associated with impeding respiratory distress. In fact, studies^38,39^ have found that clinical monitoring of oxygen saturation is not associated with early detection of alveolar hypoventilation and may therefore increase risks associated with hypoxemia and depression of respiratory activity. Five studies^40,41,42,43,44^ have suggested the utility of Petco_2_ for hemodynamic monitoring, and Petco_2_ is defined as an early warning system.^45^ In our study, Petco_2_ remained in the reference range in both groups. Additionally, PI remained in the reference range during the study period. Use of face masks among children, therefore, was not associated with alveolar hypoventilation or hemodynamic instability.

Unfortunately, 2 children in group A dropped out because they refused to wear the surgical mask. We think that this may be associated with a possible bias, evaluated in a subsequent debriefing of our research group, associated with our initial inexperience. Among the children in group A, these 2 children were the first to be tested. After this initial failure, we had the parents conduct the training of their children instead of the doctor, which was associated with better compliance.

Overall, our findings suggest that recommendations against the use of surgical masks could be reconsidered in this age group, though our findings exclude possible adverse effects associated with gas exchange in children younger 24 months wearing face masks. However, CDC and AAP guidelines^32,33^ state that infants and toddlers could have difficulty removing face masks and may not be able to communicate if they are having trouble breathing. Our findings may provide additional evidence suggesting that the use of face masks in this group of children in certain high-risk situations may not be associated with changes in respiratory function. However, constant supervision of adults would be needed.

### Limitations

This study has some limitations. The evaluation of parameters while children wore face masks was done for only 30 minutes. Further studies are needed to evaluate possible longer-term changes. Another limitation was the small sample size.

## Conclusions

This cohort study found that the use of surgical face masks among children was not associated with changes in respiratory function, including among children aged 24 months or younger. These findings may help promote the use of surgical masks among children, especially in view of the reopening of schools. Every precautionary measure against the diffusion of COVID-19 should be implemented. Furthermore, we think that children should be educated in the use of face masks by parents and school personnel. This may be associated with increased compliance with mask usage, especially among younger children. We do not know how long the present emergency will last, but we must prepare in case new lethal viruses should appear, possibly associated with increased adverse clinical outcomes among children.
